# Supplementary material for: Unraveling the mechanism of tip-enhanced molecular energy transfer
Source: Commun Chem. 2024 Feb 15;7:32. doi: 10.1038/s42004-024-01118-1 (PMC10869822; doi:10.1038/s42004-024-01118-1)
Supplement: Supplementary file 2 — Supplementary Information [file 42004_2024_1118_MOESM2_ESM.pdf]

Supplementary Information for Unraveling the Mechanism of Tip-Enhanced Molecular Energy Transfer

Colin V. Coane,[†,‡] Marco Romanelli,[†] Giulia Dall'Osto,[†] Rosa Di Felice,[*,‡,¶] and Stefano Corni[*,†,¶]

†*Department of Chemical Sciences, University of Padova, via Marzolo 1, Padova, Italy*

‡*Department of Physics and Astronomy, University of Southern California, Los Angeles, CA, 90089, USA*

¶*CNR Institute of Nanoscience, via Campi 213/A, Modena, Italy*

* E-mail: difelice@usc.edu; stefano.corni@unipd.it

Supplementary Note 1. Technical issues in evaluating $\Gamma_{\mathbf{EET}}$

Given calculations were performed using multiple different softwares, for instance Gaussian16 and GAMESS were used for evaluating V_0 and V_{met}, respectively, so care had to be taken to prevent inconsistencies. One important consideration was that transition dipoles calculated with Gaussian16 and GAMESS for the same molecule at the same donor-acceptor distance had to match in both direction and phase, so that the sum of Eq. 9 (main text) could be evaluated without any fictitious inconsistency regarding the two terms in the sum. For instance, as the first two excited states of the donor are degenerate, state ordering may swap between calculations, and so transition dipoles had to be correctly matched between softwares when combining quantities, e.g. when taking the sum V_{met} and V_0 in order not to mix up

quantities belonging to different excited states. Additionally, each dipole phase may differ up to a factor of ±1 between calculations, so a convention based on the GAMESS results was adopted and used to ensure the phases of V_0 and V_{met} matched this convention across calculations and could be added with their corresponding proper sign. This a posteriori correction was done by reversing the sign of the computed quantities when the transition dipole included an arbitrary phase swap. This correction was done for calculations at each individual distance R as phase discrepancies existed across the same calculations done at different values of R.

Supplementary Note 2. Dependence of the EET results on the tip position

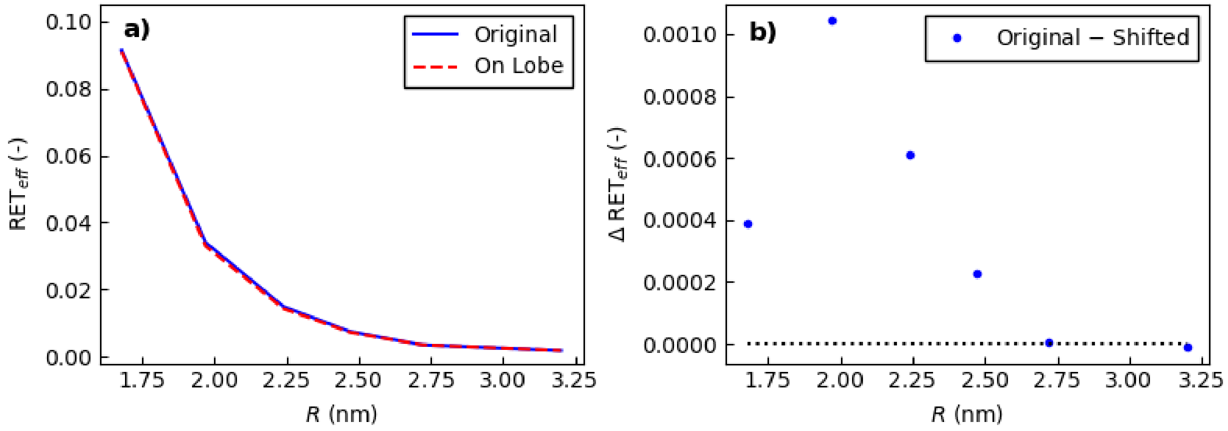

Supplementary Figure 1: a) $\mathrm{RET}_{\mathrm{eff}}$ as a function of distance between the molecule centres obtained with the tip's protrusion centre placed on the middle of the PdPc aromatic ring (solid blue line) and on the middle of the nearby lobe (red dashed line), as depicted in Figure 4a (main text) via black spots 1-2. b) Corresponding numerical difference of the simulated data of panel a).

As shown in Figure 4a (main text) two different tip positions have been considered for evaluating $\mathrm{RET}_{\mathrm{eff}}$. In one case, the tip apex is placed exactly on the middle of one of the PdPc aromatic ring, whereas in the other it is located on the centre of the nearby orbital lobe. In Supplementary Figure 1 we show the computed $\mathrm{RET}_{\mathrm{eff}}$ as a function of distance

between PdPc and H_2Pc for the two tip positions (decay rates evaluated at the original unmodified donor and acceptor frequencies), clearly showing that this spatial shift of the tip protrusion does not affect the outcome of the simulations.

Supplementary Note 3. Dependence of the donor and acceptor decay rates on the tip structure

Similar to Figure 7 (main text), plasmon-mediated decay rates are analyzed here in the case of the larger tip structure of Figure 3b (main text), as illustrated in Supplementary Figure 2. In this case, moving closer to resonance leads to a sizeable increase of both donor and acceptor radiative and nonradiative decay rates which results in a decrease of the absolute RET_{eff} value (see Figure 6 main text), in agreement with results reported in Figure 7 (main text). Nevertheless, with this tip structure radiative emission is much more enhanced than metal-induced non radiative decay, compared to results of Figure 7 (main text) where the other tip structure is used. This is mostly due to the tip's larger size, which translates into a larger NP-induced dipole moment that sizably contribute to the radiative rate expression of Eq. 12 (main text). Indeed, this tip structure was previously shown to considerably boost photoluminescence emission of single molecules, making TEPL experiments able to disclose sub-molecular features.[1,2]

In this context, the plasmon modes of such tips that most efficiently couple with molecules are those featuring charge localization at the tip apex. Previous works have shown that the spatial electric field distribution over the molecular plane is much affected by the apex geometrical features, thus corroborating that tip geometrical features can drastically affect plasmon-mediated molecular properties.[1–3]

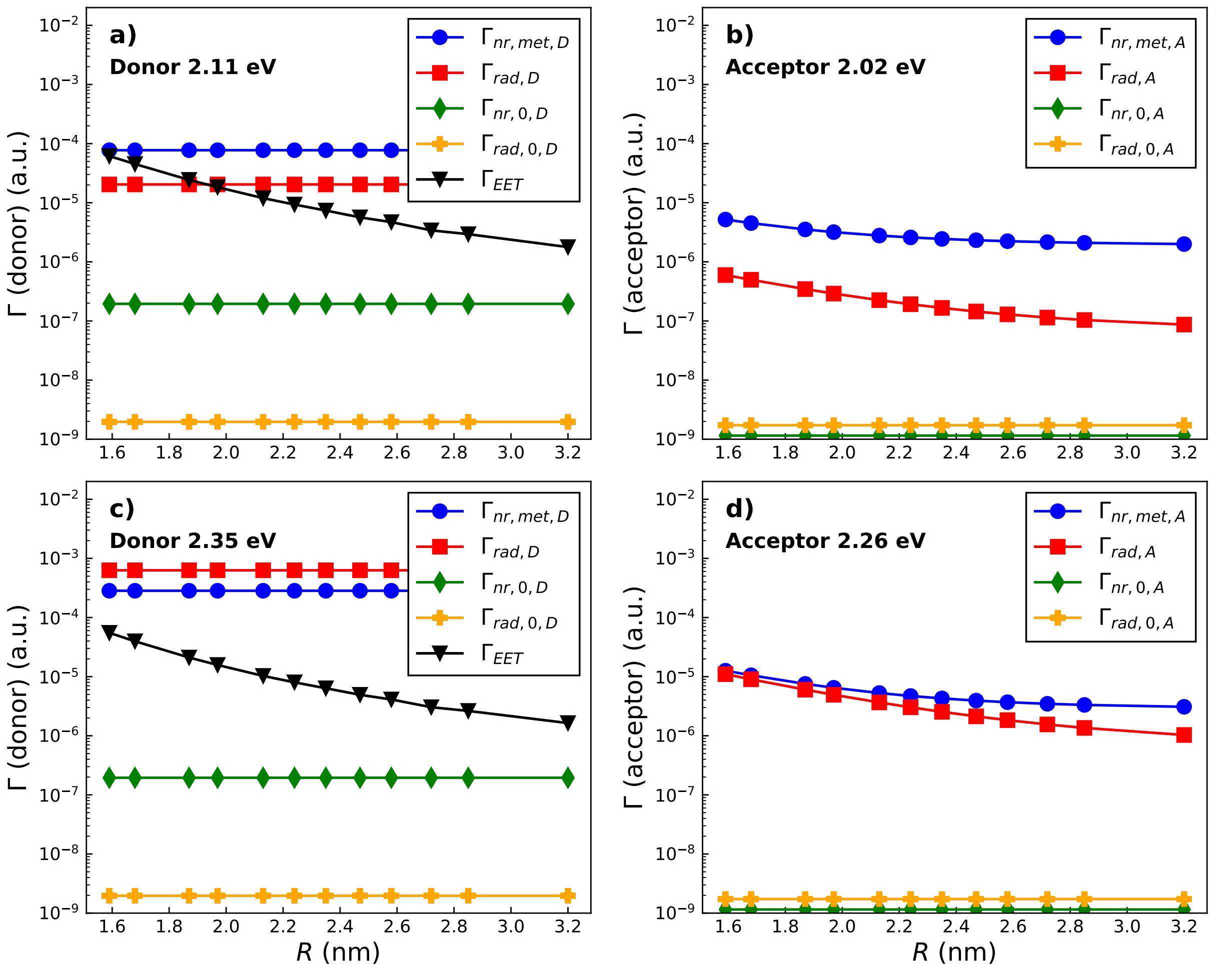

Supplementary Figure 2: Comparison of donor and acceptor S_1 states decay rates that contribute to the EET efficiency, computed as in Eq. 7, as a function of donor-acceptor distance in a logarithmic scale in the presence of the tip structure of Figure 3b (main text).The metallic response affecting the different rates has been evaluated at the respective donor and acceptor excitation frequencies ($\omega_D \approx 2.11$ eV and $\omega_A \approx 2.02$ eV, panels a and b, respectively) and with the donor frequency shifted to the tip's resonance peak energy ($\omega_D = 2.35$ eV) while keeping the same difference between donor and acceptor $\omega_{DA} \approx 0.09$ eV (panels c and d, respectively). Panels a and c show the nonradiative decay rate of the donor induced by the metal ($\Gamma_{nr,met,D}$, blue line), the radiative decay rate of the donor in the presence of the metal ($\Gamma_{rad,D}$, red line), the intrinsic nonradiative decay rate of the donor ($\Gamma_{nr,0,D}$), the intrinsic (vacuum) radiative decay rate of the donor ($\Gamma_{rad,0,D}$) and the metal-mediated electronic energy transfer rate (Γ_{EET},black line). Panels b and d show the corresponding quantities for the acceptor molecule (excluding the EET rate), evaluated at the acceptor frequency.

Supplementary Note 4. Dependence of EET results on tip-molecule distance

All results reported in main text are obtained with a fixed tip-molecule distance for a given tip setup. Increasing the tip-molecule distance leads to an overall increase of $\mathrm{RET_{eff}}$ as illustrated in Supplementary Figure 3. This result is in agreement with what is observed in Figure 6 (main text) moving out of resonance. Indeed, in that case the absolute value of $\mathrm{RET_{eff}}$ increases because of smaller plasmon-mediated radiative and non-radiative decay rates that enter into the denominator of Eq. 7 (main text). Enlarging the molecule-metal separation also leads to a similar trend as plasmon effects on molecular decay rates progressively attenuate because the mutual interaction gets weaker.

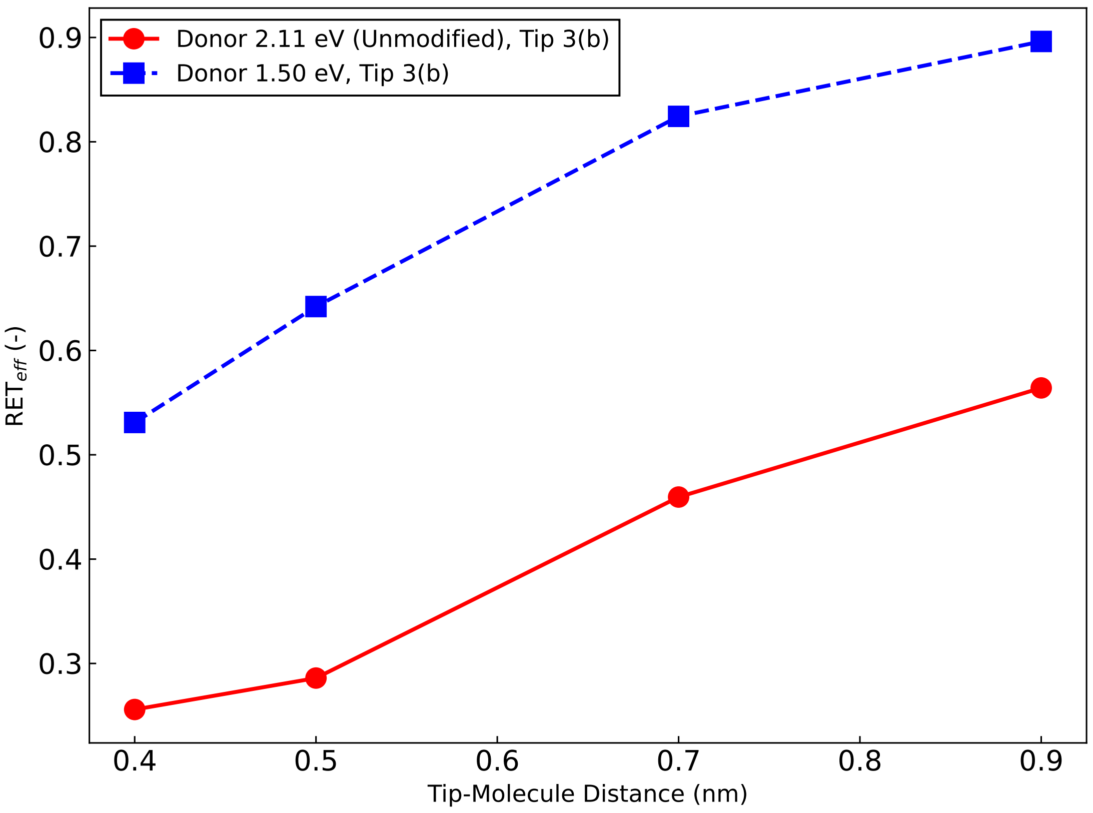

Supplementary Figure 3: Comparison of $\mathrm{RET_{eff}}$ as a function of tip-donor distance obtained using the tip structure of Figure 3b, and evaluated at the donor absorption frequency of 2.11 eV (unmodified donor frequency, red line), and with absorption frequency shifted to 1.50 eV (blue line). The donor-acceptor separation is kept fixed at ≈ 2 nm.

Supplementary Note 5. Dependence of EET results on spectral overlap between PdPc and H_2Pc S_2 state

The spectral overlap value entering into Eq. 9 is set to the experimental[4] value of 1.4 eV^{-1} when the S_1 state (Q_x band) of H_2Pc is involved, whereas the corresponding experimental value for the S_2 state (Q_y band) is missing, since its contribution to the spectral overlap in ref.[4] has been neglected. All results reported in the main text consider the same J value for both states, as sizable changes of the spectral overlap values for the S_2 state contribution do not lead to qualitative differences in the RET_{eff} decay trend, as shown in Supplementary Figure 4.

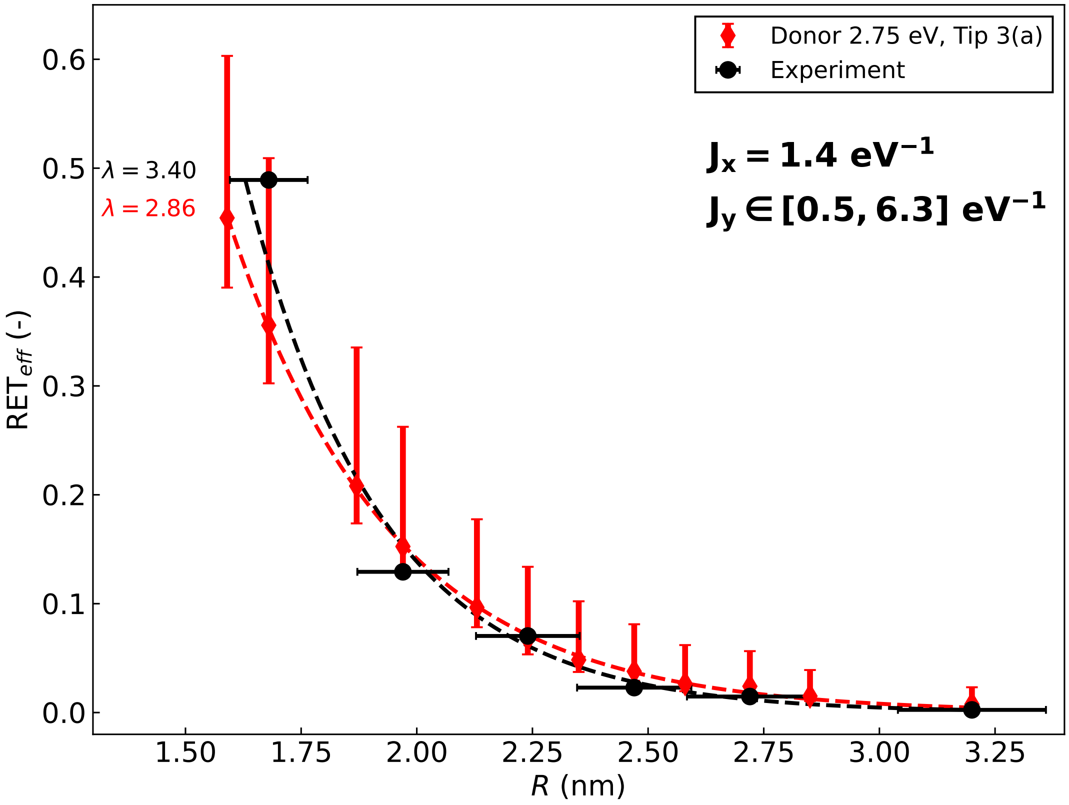

Supplementary Figure 4: RET_{eff} as a function of donor-acceptor distance obtained using the tip structure of Figure 3a, and evaluated with the donor absorption frequency set to 2.75 eV. The spectral overlap value is set to the experimental value of 1.4 eV^{-1} for the S_1 state of H_2Pc (J_x), while the error bars of the J value for the S_2 state (J_y) span the range $0.5 - 6.3\,eV^{-1}$. The lower (upper) extreme of each red bar represents the corresponding RET_{eff} value computed using $J_y = 0.5\,eV^{-1}$ ($J_y = 6.3\,eV^{-1}$).

Supplementary References

(1) Yang, B.; Chen, G.; Ghafoor, A.; Zhang, Y.; Zhang, Y.; Zhang, Y.; Luo, Y.; Yang, J.; Sandoghdar, V.; Aizpurua, J.; others Sub-nanometre resolution in single-molecule photoluminescence imaging. *Nature Photonics* **2020**, *14*, 693–699.

(2) Romanelli, M.; Dall'Osto, G.; Corni, S. Role of metal-nanostructure features on tip-enhanced photoluminescence of single molecules. *The Journal of Chemical Physics* **2021**, *155*, 214304.

(3) Doppagne, B.; Neuman, T.; Soria-Martinez, R.; López, L. E. P.; Bulou, H.; Romeo, M.; Berciaud, S.; Scheurer, F.; Aizpurua, J.; Schull, G. Single-molecule tautomerization tracking through space-and time-resolved fluorescence spectroscopy. *Nature nanotechnology* **2020**, *15*, 207–211.

(4) Cao, S.; Rosławska, A.; Doppagne, B.; Romeo, M.; Féron, M.; Chérioux, F.; Bulou, H.; Scheurer, F.; Schull, G. Energy funnelling within multichromophore architectures monitored with subnanometre resolution. *Nature Chemistry* **2021**, *13*, 766–770.
