# Supplementary material for: Unraveling the mechanism of tip-enhanced molecular energy transfer
Source: Commun Chem. 2024 Feb 15;7:32. doi: 10.1038/s42004-024-01118-1 (PMC10869822; doi:10.1038/s42004-024-01118-1)
Supplement: Supplementary file 3 — Description of Additional Supplementary Files [file 42004_2024_1118_MOESM3_ESM.pdf]

Description of Additional Supplementary Files

File name: Supplementary Data 1

Description: Source data of main text figures

File name: Supplementary Data 2

Description: Atomic coordinates of PdPc and H2Pc molecular structures
